# Perceptions of nursery staff and parent views of healthy eating promotion in preschool settings: an exploratory qualitative study

**DOI:** 10.1186/s12889-016-3507-x

**Published:** 2016-08-19

**Authors:** Lorraine A. McSweeney, Tim Rapley, Carolyn D. Summerbell, Catherine A. Haighton, Ashley J. Adamson

**Affiliations:** 1 Institute of Health & Society, Human Nutrition Research Centre, Newcastle University, Newcastle upon Tyne, UK; 2 Institute of Health & Society, Newcastle University, Newcastle upon Tyne, UK; 3 School of Medicine, Pharmacy and Health, Durham University, Durham, UK

**Keywords:** Preschool children, Nursery schools, Preschools, Parents, Obesity, Healthy eating, Policy implementation, Health promotion, Qualitative

## Abstract

**Background:**

In the UK just over a fifth of all children start school overweight or obese and overweight 2–5 year olds are at least 4 times more likely to become overweight adults. This can lead to serious future health problems. The WHO have recently highlighted the preschool years as a critical time for obesity prevention, and have recommended preschools as an ideal setting for intervention. However, existing evidence suggests that the preschool environment, including the knowledge, beliefs and practices of preschool staff and parents of young children attending nurseries can be a barrier to the successful implementation of healthy eating interventions in this setting.

**Methods:**

This study examined the perceptions of preschool centre staff and parents’ of preschool children of healthy eating promotion within preschool settings. The participants were preschool staff working in private and local authority preschool centres in the North East of England, and parents of preschool children aged 3–4 years. Preschool staff participated in semi-structured interviews (*n* = 16 female, 1 male). Parents completed a mapping activity interview (*n* = 14 mothers, 1 father). Thematic analysis was applied to interpret the findings.

**Results:**

Complex communication issues surrounding preschool centre dietary ‘rules’ were apparent. The staff were keen to promote healthy eating to families and felt that parents needed ‘education’ and ‘help’. The staff emphasised that school policies prohibited providing children with sugary or fatty snacks such as crisps, cakes, sweets and ‘fizzy’ drinks, however, some preschool centres appeared to have difficulty enforcing such guidelines. Parents were open to the idea of healthy eating promotion in preschool settings but were wary of being ‘told what to do’ and being thought of as ‘bad parents’.

**Conclusions:**

There is a need to further explore nursery staff members’ personal perceptions of health and how food policies which promote healthier food in preschool settings can be embedded and implemented. Family friendly healthy eating strategies and activities which utilise nudge theory should be developed and delivered in a manner that is sensitive to parents’ concerns. Preschool settings may offer an opportunity for delivery of such activities.

## Background

Globally in 2013 the number of overweight children under the age of 5 years was estimated to be over 42 million [1]. In the UK around a fifth (22.5 %) of young children start school overweight or obese [2] and the prevalence is greatest in young children from low socioeconomic and ethnic minority backgrounds [3]. Using the UK90 clinical cut points [4] an overweight child is classified as ≥91^st^ centile and an obese child ≥98^th^ centile. Overweight 2–5-year-olds are at least 4 times more likely to become overweight adults than normal weight children [5]. Long-term health risks of overweight and obesity include: obesity persistence; cardiovascular risk factors; stroke; gall bladder disease; diabetes; fatty liver disease; and some cancers [6–8]. The WHO Commission on Ending Childhood Obesity report that progress in tackling childhood obesity has been slow and inconsistent and the preschool years have been highlighted as a critical period for obesity prevention [3]. A recent systematic review found that preschool practitioners’ practices are associated with young children’s eating behaviours [9], and the potential of preschool centres as a setting for influencing young children’s food choice has been critically reviewed [10].

The UK has become the highest spender on early year’s services in Europe [11]. In 2015 94 % of 3-year olds and 99 % of 4-year olds benefited from some form of free early education at maintained schools or in the private, voluntary or independent sector [12]. Early years providers in England follow the mandatory Framework for the Early Years Foundation Stage (EYFS) [13]. One component of the EYFS educational programme that must be implemented is ‘Physical development’ within which, ‘children must be helped to make healthy choices in relation to food’ [13] (p.5). It follows then, that preschool settings may provide valuable opportunities to access children and their families not only for promoting healthy lifestyles, but also to develop and evaluate behaviour-change interventions [14–16] which would contribute to the aim of reducing health inequalities. Traditionally, parents have had the dominant role for determining eating behaviours in their preschool child, however, as the statistics show, child-care providers may also have a primary role in influencing children’s eating behaviours [17, 18]. The importance of preschool settings in the UK has been recognised in the NICE obesity guidelines [19].

There is a body of evidence which suggests that practitioner and parental involvement is key for the success of an intervention aimed at obesity prevention in children [20, 21]. Baranowski, Cerin [22] highlights the need to ensure interventions meet the needs and capitalise on the strengths of their respective target population and that evidence is practicable in the ‘real world’; that is, interventions should target strategies and techniques which aid parents in modifying their child’s diet [23]. Research suggests that whilst parents understand *what* they should be feeding their children, they do not always know *how* to [24]. Furthermore, preschool practitioners may be unaware of the impact of their modelling behaviours and some negative feeding practices have also been reported [9]. Whilst there have been numerous obesity prevention interventions conducted in primary schools [25], there is a paucity of interventions targeting children in preschool settings in the UK. Before a behaviour-change intervention can be designed and implemented, it is important to gain an in-depth understanding of parents and staff insights and knowledge of everyday practices in preschool settings [26]; the best way to achieve this is via qualitative methods. This study aimed to assess the knowledge, beliefs and practices of preschool staff and parents of young children attending a preschool centre about the value and need for healthy eating promotion in preschool settings and identify any issues which they feel need to be taken into consideration when developing interventions.

## Methods

### Study design

Explorative, qualitative design.

### Participants and recruitment

Participants were qualified nursery staff (teachers, nursery practitioners, nursery nurses or early years’ workers) working in private and local authority preschool centres in the North East of England, and parents of preschool children aged 3–4 years. A convenience sample was selected for the study. Forty preschool centres were contacted by invitation letter and follow-up telephone call; five head-teachers consented for their preschool centre to take part and provided contact details of interested members of staff; 17 staff members in total consented to participate. Preschool staff distributed study information to parents and provided contact details of those interested; following a telephone conversation, 14 mothers and one father consented to take part.

### Semi-structured interviews

Semi-structured interviews were selected for gathering healthy eating promotion information from the nursery staff as they enable a focused and in-depth exploration of topics [27]. They combined a mix of ‘closed’ and ‘open’ ended questions, although the questions were planned, they were designed to be flexible [28], thus allowing narratives of the interviewees to flow and expand, which may add further unplanned dimensions to the topics. The nursery staff interviews lasted approximately 30 min and were all conducted on an individual basis at the staff member’s place of work. The interviews were conducted by the lead author, who has a post-doctoral degree and previously worked as a nursery practitioner, using a topic guide which was developed from the literature (available on request from the author LM). The semi-structured interview questions were designed to incorporate a range of issues, such as: “Who is responsible for ensuring children follow a healthy lifestyle?” Additionally, to elicit departmental practices and policies, questions such as: “What is the nursery’s practice regarding fizzy drinks, sweets, crisps and so forth?” Finally, questions of a more personal nature were asked, such as, “Do you think your own actions and views on diet and healthy living have an impact on the children in your care?” The topic guide and parent mapping activity were pilot-tested by a small group of university parents who had or previously had a preschool-aged child attending a preschool centre; this was essentially to test the topic guide and the data was not included in the analysis. LM guided the interviews using the topic guide and interviews were audio recorded with the participant’s written informed consent.

### Mapping activities

**Fig. 1:**
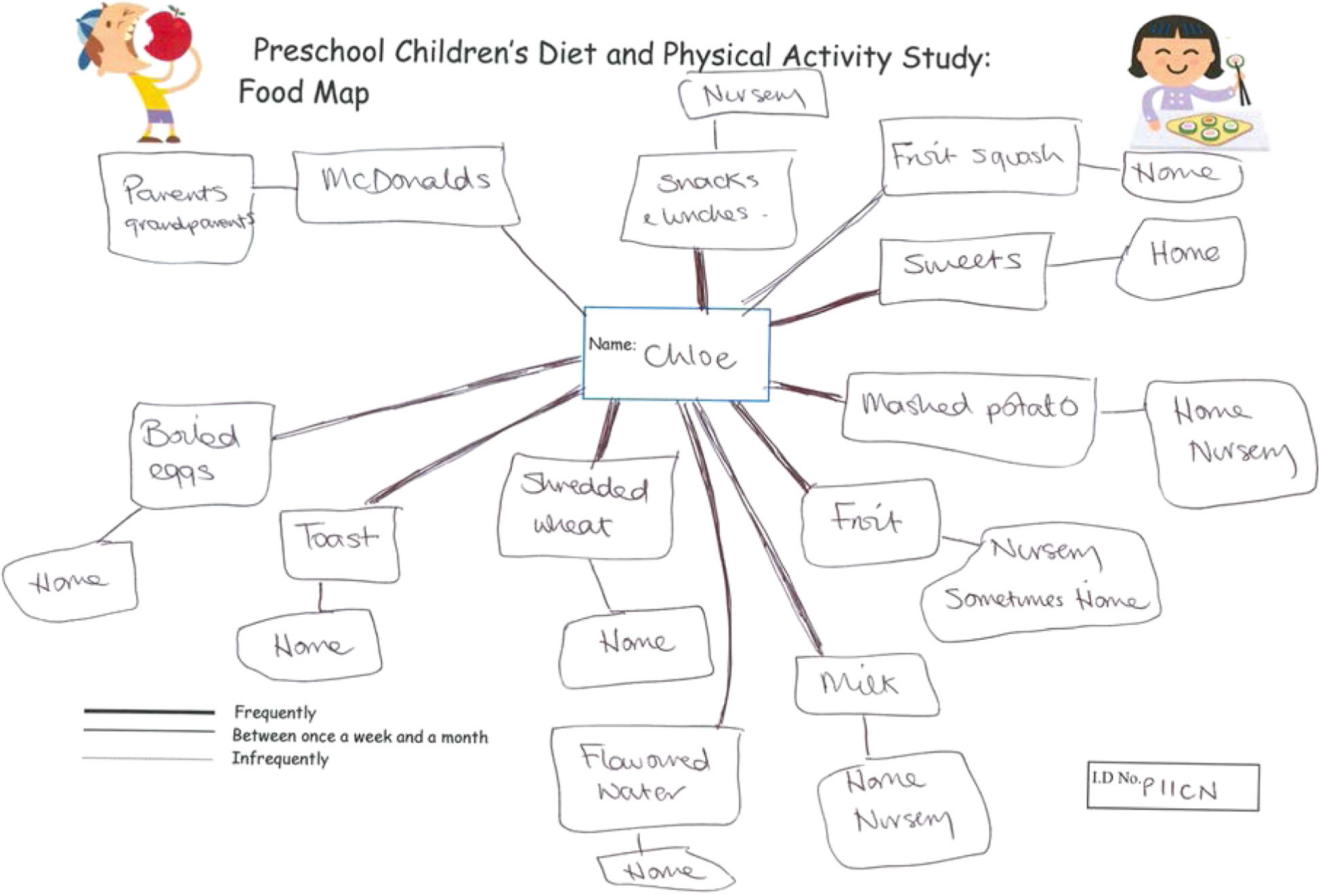
Example of child’s completed food map

Parents completed a food map as described by Albon [29] (Fig. 1) which offers a useful visual representation of their child’s current dietary behaviour. The mapping exercise, which lasted approximately 30 min, took place with the lead author either at the child’s preschool centre or at the parent’s home. The mapping exercise also acted as a tool to elicit further discussion about family and preschool centre healthy eating practices. The mapping discussions were audio recorded with the participant’s written informed consent.

### Data management and analysis

The interviews and mapping activities were transcribed verbatim and stored securely. Thematic analysis [30] was adopted to identify themes. NVivo software was used to aid indexing and charting [31]. Guided by the principles of grounded theory [32], the data were repeatedly read and coded independently by researcher LM within a framework of a priori issues identified from the topic guide and by participants or which emerged from the data. Analysis was discussed at regular meetings with TR, an experienced qualitative researcher to identify areas for closer consideration and to enhance credibility of the thematic analysis and interpretation. The participants were given identity codes which describes role (staff/teacher/parent) and type of nursery; any names used are pseudonyms.

### Ethics statement

The study was reviewed and granted ethical approval by Newcastle University’s Faculty of Medical Science ethics committee (00456/2011).

## Results

Seventeen nursery staff members, (Table 1) and 15 parents (Table 2) consented to take part in interviews or the mapping exercise interview; total numbers of available staff members or parents for participation were not known, however, we estimate 30 staff and 250 parents. Analysis and interpretation of the interviews and mapping activity dialogue revealed many overlapping themes and issues; therefore the results from staff and parents are presented and discussed together.

**Table 1:** Age and work experience of the 17 preschool staff who were included in the interviews for this study

Age group	Number	Years qualified	Number	Years of service	Number
16–25	2	1–4	6	1–4	5
26–35	2	5–9	3	5–9	5
36–45	8	10–14	3	10–14	2
46–55	4	15–20	3	15–19	2
Over 55	1	20 or more	2	20 or more	3

**Table 2:** Socio-demographic indicators of the 15 parents who were included in the mapping activity for this study, and the characteristics of their children

Age group	Marital status	Level of education	Gender of child	Age of child
16–25	Single	Some secondary school	Female	3
16–25	Living with a partner	Completed secondary school	Female	3
16–25	Living with a partner	Some additional training	Female	4
26–35	Separated	Some additional training	Male	3
26–35	Married for the first time	Undergraduate university	Male	4
26–35	Single	Completed secondary school	Female	4
26–35	Single	Some additional training	Female	4
26–35	Separated	Some additional training	Female	4
26–35	In a steady relationship	Some additional training	Male	4
26–35	Single	Some additional training	Male	4
36–45	Married for the first time	Some additional training	Female	4
36–45	Married for the first time	Undergraduate university	Male	3
36–45	Married for the first time	Some additional training	Male	4
36–45	Married for the first time	Undergraduate university	Female	4
36–45	Living with a partner	Completed secondary school	Female	4

Parents were more likely to report their child’s favourite food was fruit and vegetables over other types of foods. Debbie, a mother of a 3-year-old who attended a local authority preschool, was keen to emphasise the positive aspects of her son’s diet:

I’m trying to stick to the healthy stuff, so it doesn’t sound as bad. (P1LA1).

Once the mother ascertained that it was acceptable to talk about other aspects of her child’s diet, she was more open and reported more about her family’s diet:

Yeah oh, liquorice, he loves his liquorice, all the usual rubbish, pizza….. Oh, as regular as everything else, we’re terrible..... (P1LA1).

Some parents may have felt it was important to present themselves in a favourable light when discussing their child’s diet and health. Nursery staff were asked to give their views on where they believed the responsibility lay for ensuring that children led a healthy lifestyle. Several nursery staff members were eager to stress that they did not directly blame parents for unhealthy behaviours, however, some statements suggested otherwise:

There are young parents who don’t know… who need parenting skills on how to feed their children properly… they’ve got to be taught how (T2LA1).

Society, busy lifestyles and having to work were stated as impeding parents’ abilities to maintain their child’s health and/or to become involved in ventures that would assist them to do so. Although the views of the staff may have been reactive, some strong opinions were expressed. Some examples focused on the types of foods children were provided with by their parents which nursery staff classed as ‘unhealthy’ foods:

Children come into nursery and they’re eating crisps or sweets and they’re met at the end of the day with sweets as well (STAFF1).

This may have been expressed as a statement that the parental practices contravened preschool ‘rules’. A community nursery worker was keen to differentiate the preschool and parental feeding practices:

We wouldn’t give them stuff, obviously, that the parents do give them (STAFF14).

The use of the word ‘obviously’ suggests that preschool practices were regarded as desirable and that the parents perhaps provided food regarded as unhealthy.

An overriding theme that emerged from the data was the staff members’ interpretation that parents required ‘help’ and ‘education’ with their child’s health and that parents’ perceived lack of knowledge was a barrier. One community nursery worker expressed her feelings:

….but if the parents aren’t educated in healthy eating, someone has got to intervene to show them how to… (STAFF14).

The idea that preschool centres could offer healthy eating advice to families produced mixed reactions from parents. It was agreed that preschools were an ideal base for providing information but how it was delivered to parents produced much discussion.

….because if you say to children, “Right, this is really important,” then they may go home and tell their parents, but that’s not going to just make any difference, or a letter home to parents saying, “Don’t let your child do this,” they’re just going to not really take any notice (P3LA2)

The belief that preschool centres could just provide information or send information home with the child was deemed to be impractical and futile. However, one parent thought providing information would be useful:

[parents]… should have loads of information, like a little push into the right direction of how kids should be…(P1LA2).

Encouraging parents to actively engage in preschool activities was suggested by one mother to be the most efficient way of promoting change. However, there was repeated concern about being ‘told what to do’ and the view that some parents may not want to change lifestyle behaviours or feel they are being told that they are ‘bad’ parents. One mother to a four-year-old boy, wanted to emphasise a more positive approach:

…. doing it in a positive way, not just saying, you mustn’t do this, you mustn’t do this, otherwise you’re bad parents (P2LA1).

An interesting aspect that emerged from some parents was the overall sense that they were talking about *other* parents and not themselves;

Because so many people don’t know how to cook, you know, adults don’t know how to cook these days…and so the future generation needs to kind of learn those things (P5LA2).

It became a very much “them and us” type of conversation. There appeared to be an issue with potential barriers to healthy eating occurring not only in the home as suggested by preschool staff but also within the preschool environment. Conflicting opinions arose from a lack of standardised guidelines. However, the majority of the staff contributing their views did believe that the preschool centres were setting good examples. Some parents demonstrated a lack of knowledge as to what types of foods were being provided to their children in the preschool setting, one mother was uncertain;

I’m not too sure what they have on. I know they have… they do fruit don’t they? I think they do tea cake. I don’t know what he actually has (P1LA1).

Parents whose children stayed for a full-day session at nursery were more likely to have a better idea of what their child received and were positive about what was provided;

…he does eat things he wouldn't normally eat at home… that he refuses at home he’ll eat at nursery (P1LA1).

However, overall, conflicting messages appeared to be conveyed to parents about preschool dietary ‘rules’; especially in the area of ‘treats’ and birthday celebrations. Preschool staff made it clear that school policies prohibited providing children with sugary or fatty snacks such as crisps, cakes, sweets and ‘fizzy’ drinks. However, there appeared to be inconsistencies in enforcing this even among staff belonging to the same preschool centre. Most centres discouraged parents providing birthday cakes. In all but one private preschool there appeared to be difficulty in enforcing the ban and sometimes cakes, party bags or snacks were provided, as one nursery staff member explained:

Sometimes parents can bring in a birthday cake or they might bring in snack, they do tend to be cake and crisps…..we might add fruit to it. (STAFF17).

The nursery staff member from this local authority preschool centre appeared unhappy with the current preschool practices, adding fruit to the ‘unhealthy’ snacks may have been a way to assuage any guilt in breaching the preschool practices or she may have felt it was just the ‘right’ thing to do. Some centres did tolerate the provision of cakes and sweets from parents despite stating in the interviews that the preschool centre’s healthy eating policy did not allow such foods within the preschool environment. Therefore, the messages portrayed to parents appeared to be contradictory. A common way to ‘deal’ with the issue in several centres was to place the responsibility of passing the child’s ‘treat’ onto the parents, as a staff member from a private preschool explained:

We cut it up (cake) and let them take it home … it’s up to the parents what they want to do … same with sweets and stuff as well … just send it home, we haven’t got … we never allow them in here no, just send them home (STAFF11).

Some mothers gave the impression that they had not really considered the provision of birthday cakes to be an issue; again, this could be linked to the cultural norm of celebrating special occasions with food. Once the mothers ascertained that it could be a frequent occurrence within their child’s class, they seemed torn in their opinions:

I think it’s easier for the nursery just to say “none” rather than to be the arbiter of what is healthy and what isn’t, because then you’re going to have the situation where a parent arrives with a cake, and the nursery staff might say, “Well, actually, that doesn’t fall into our list of ‘healthier’ cakes, so you can’t bring it.” So it just does away with all of that, it just says “none” (P4LA1)

These conflicting practices and messages may be due to the difficulty in enforcing an ‘all out ban’ in the ‘real’ world where food and ‘treats’ are so abundant, available and integrated in daily contemporary life and part of food culture. When asked how nurseries might enforce an all-out ban on high sugar, salt and fat snacks one nursery staff member admitted that imposing such a policy could be difficult:

For the sugar and that they very rarely get, it’s Christmas time or if the parents bring a bag in with sweets we let them take home. We don’t actually have them at nursery. It is going to be hard, it will be hard (STAFF12).

It is interesting to note that some staff criticised parents for being unable to impose ‘healthy eating’ practices with their children and for ‘giving in’. However, it seems, preschools too have difficulty in enforcing or communicating regulations by allowing the parents to provide excluded foods in the preschool environment.

## Discussion

This study enabled the perceptions of preschool staff and parents with regards to healthy eating promotion in preschool centres to be compared. An overarching theme that emerged was the concern of preschools finding the right balance of communicating healthy eating information and advice without patronising or telling parents how to raise their children. Parents in this study reported being happy to receive healthy eating advice from their child’s preschool but did not want to be ‘told what to do’. As reported in findings from the Europe-wide Toybox Study, simply providing parents with knowledge and information through letters and newsletters is not adequate for intervention [33]. This presents the challenge; how to ensure that nursery staff are promoting the same (ideal) messages without parents feeling they are being patronised.

A systematic review conducted in 2010 reported that parents felt professionals blamed them for their child’s weight problems [34]; this may extend to other health issues such as healthy eating. Nursery staff and parents felt that preschools did have a responsibility to promote healthy eating to young children. However, as suggested in this study, promoting positive health behaviours to families of young children is a complicated issue. The preschool staff had preconceived ideas about healthy eating and what constituted a healthy lifestyle. A recent qualitative study conducted with nursery workers in Liverpool demonstrated that there are many complex issues which may hinder or support preschool centres to develop a healthy eating culture [35]. Indeed, an unexpected finding from the present study was the apparent difficulty or intentional lack of compliance with preschool health policies such as the permitting of high energy dense snacks and foods. Although preschool centres have policies advocating healthy practices within the preschool setting, how well these are communicated to parents is not known. As was reported above, some parents were unaware of the types of food being offered to their child, so may also not be aware of preschool eating policies.

The parents and staff had their own preconceived ideas of what it meant to be ‘healthy’. Indeed it has been reported that child care professionals hold misconceptions with regards to child feeding and prevention of obesity [36]. Furthermore, it is reported that increased knowledge, curriculum requirements and resources does not automatically equate to child care providers changing their own behaviours or beliefs [36].

The staff believed that parents needed help and education, conversely the parents in the study believed that *other* parents needed help and education. This perhaps suggests that a person’s own ‘norm’ is acceptable but others are not.

The majority of the staff indicated that they were happy to promote health and give advice to parents. However, the understanding of what this might entail is uncertain. A recent qualitative study examining preschool nutrition and policy concluded that preschool centres are genuinely interested in implementing healthy eating policies but need further support to achieve this [37]. Similarly, although the staff in the present study reported that they believed parents needed help and educating about their children’s health, they were unable to detail where this help would come from and how it would be best delivered.

The use of ‘nudge theory’, which has gained particular momentum in areas such as health promotion [38], may be a tool which can be utilised by preschool settings to facilitate the promotion of healthy eating to families in a non-paternalistic way. A nudge is described as ‘an aspect of the choice architecture that alters people’s behaviour in a predictable way without forbidding any option or significantly changing their economic incentives’ ([39], p. 6). A nudge can involve making an environment less conducive to someone making an unhealthy choice; provision of information; changes to default; and the use of norms [40]. It could be argued that the member of staff in this study who provided fruit with the birthday cake was performing a nudge, in that the children had a healthy option easily available to them.

A recent systematic review by Mikkelsen, Husby [10] concluded that preschools are potentially important settings for influencing young children’s dietary behaviours but further research in this area was still required. In order to develop a healthy eating intervention involving preschool children and their families, it is recommended that staff and parents should be involved in the design of the intervention, with appropriate training and support given. The need to ensure that the evidence base is driven by user involvement is a high priority [41]. ‘Family friendly’ activities and strategies should be developed and delivered in a manner that is sensitive to the parents’ concerns of ‘being told what to do’. This is where nudge theory could be utilised.

### Future research

There is a need to further explore preschool staff members’ personal perceptions of health and how food policies which promote healthier food in preschool settings can be embedded and implemented [42] as curriculum requirements do not always guarantee staff compliance [36]. Moreover, how these policies can best be communicated to parents and families and how staff would like parents to comply with these types of policies also warrants further investigation. As previously discussed, nudge theory may be a tool which can be incorporated into future preschool interventions.

### Strengths and limitations

The study is strengthened by having the perspective of both parents (although only one father) and preschool staff. However, time and project funding did not allow for validation of the findings with the participants. Additionally, it should be noted that the lead researcher’s previous occupation as a nursery practitioner may have introduced some bias into the interpretation of the findings.

## Conclusions

This study shows that both preschool staff and parents believe healthy eating promotion within preschool settings is important for young children’s health and education. It was reported that all the preschool centres followed healthy eating policies and guidelines. However, policies were not always observed, or well communicated leading to some conflicting practices. Staff felt parents needed ‘help’ and ‘education’ with their child’s eating habits, yet parents were wary of ‘being told what to do’. Whilst the issues of the prevention of childhood obesity continues to be challenging, the preschool years may provide a crucial window of opportunity for intervention. Given that 99 % of UK 4-year-olds access preschool settings, the potential gain is high.

## Abbreviation

EYFS: Early Years Foundation Stage
